# Modeling the impact of COVID-19 on Retina Clinic Performance

**DOI:** 10.1186/s12886-021-01955-x

**Published:** 2021-05-10

**Authors:** Karan Sethi, Emily S. Levine, Shiyoung Roh, Jeffrey L. Marx, David J. Ramsey

**Affiliations:** 1 Tufts University School of Medicine, Boston, Massachusetts USA; 2 Lahey Hospital & Medical Center, Peabody, Massachusetts USA

**Keywords:** COVID-19, Health Services Research, Discrete‐event simulation, Patient flow, Outpatient

## Abstract

**Background:**

COVID-19, a highly contagious respiratory virus, presents unique challenges to ophthalmology practice as a high-volume, office-based specialty. In response to the COVID-19 pandemic, many operational changes were adopted in our ophthalmology clinic to enhance patient and provider safety while maintaining necessary clinical operations. The aim of this study was to evaluate how measures adopted during the pandemic period affected retina clinic performance and patient satisfaction, and to model future clinic flow to predict operational performance under conditions of increasing patient and provider volumes.

**Methods:**

Clinic event timestamps and demographics were extracted from the electronic medical records of in-person retina encounters from March 15 to May 15, 2020 and compared with the same period in 2019 to assess patient flow through the clinical encounter. Patient satisfaction was evaluated by Press Ganey patient experience surveys obtained from randomly selected outpatient encounters. A discrete-events simulation was designed to model the clinic with COVID-era restrictions to assess operational performance under conditions of increasing patient and provider volumes.

**Results:**

Retina clinic volume declined by 62 % during the COVID-19 health emergency. Average check-in-to-technician time declined 79 %, total visit length declined by 46 %, and time in the provider phase of care declined 53 %. Patient satisfaction regarding access nearly doubled during the COVID-period compared with the prior year (*p* < 0.0001), while satisfaction with overall care and safety remained high during both periods. A model incorporating COVID-related changes demonstrated that wait time before rooming reached levels similar to the pre-COVID era by 30 patients-per-provider in a 1-provider model and 25 patients-per-provider in a 2-provider model (*p* < 0.001). Capacity to maintain distancing between patients was exceeded only in the two 2-provider model above 25 patients-per-provider.

**Conclusions:**

Clinic throughput was optimized in response to the COVID-19 health emergency. Modeling these clinic changes can help plan for eventual volume increases in the setting of limits imposed in the COVID-era.

**Supplementary Information:**

The online version contains supplementary material available at 10.1186/s12886-021-01955-x.

## Introduction

Severe acute respiratory syndrome coronavirus 2 (SARS-CoV-2), a highly contagious respiratory virus, presents unique challenges to ophthalmology practice [1–3]. Ophthalmologists operate in close physical proximity to their patients because of the nature of the eye examination, putting providers at relatively greater risk of exposure to the virus that causes coronavirus disease 2019 (COVID-19). Additionally, ophthalmology is typically a high-volume, office-based specialty, adding to the risk of transmission.

On March 15, 2020, the state of Massachusetts issued a stay-at-home order in an effort to control the spread of COVID-19 [4]. Despite this directive, Massachusetts was heavily affected by the pandemic. It had the third highest mortality and number of cases per capita across all states during the spring of 2020 [5, 6]. In response to the stay-at-home order, many operational changes were adopted in our ophthalmology clinic to enhance the safety of patients and providers, while maintaining necessary clinical operations. Foremost was an immediate reduction in clinic volume. Only emergency and urgent visits took place during this period, such as for those patients with conditions that required time-sensitive and necessary in-person management [7]. Patients with non-urgent conditions were either offered telemedicine or were rescheduled, as appropriate. Our office was reconfigured and waiting room capacity was limited to promote physical distancing. Protective barriers were installed, and additional time was added for staff to clean surfaces. These enhanced safety protocols were on par with those enacted by other ophthalmology and retina clinics [8–10]. Finally, to decrease the amount of time that patients spent in the office, the eye exam was streamlined, and ancillary testing was either reduced or performed at a different time [1], similar to strategies implemented by other ophthalmology clinics in order to see patients in person only when critically necessary [11, 12].

This paper systematically evaluates the impact of these COVID-related operational changes on our retina clinic by evaluating electronic medical record (EMR) timestamps related to patient flow through the clinical encounter. This analysis was supplemented by a chart review of clinical events related to the delivery of retina care. We also assessed the impact of these operational changes on our patients’ satisfaction with their care by comparing patient experience surveys during the pre-COVID and COVID eras. Finally, we used these COVID-era metrics as variables in a model to simulate clinic flow with the objective of predicting operational performance under conditions of increasing patient and provider volumes. We specifically evaluated two related areas of clinic performance: patient safety, defined as the ability to maintain physical distancing and operate within clinic-defined capacity, and clinic performance, reflected by average patient wait time, total visit length, and duration of a clinic day required to care for all patients.

## Methods

This research followed the tenets of the Declaration of Helsinki and was approved as a quality improvement initiative by the institutional review board of the Lahey Hospital & Medical Center. Information was gathered and secured in compliance with the Health Insurance Portability and Accountability Act. The requirement for informed consent was waived because of the retrospective nature of the study.

### Electronic health record data extraction

An analysis was made of eight weeks’ worth of electronic medical records (EMR) from our academic multispecialty group practice facilities spanning the dates of March 15 to May 15 in 2019 and 2020. These dates were chosen to cover the period from the Massachusetts stay-at-home order, which took effect Monday, March 16, 2020 through the peak number of daily COVID-19 cases on April 17, 2020, as well as the period in May when cases in Massachusetts began to decline [5]. EMR-based events for all patients identified during this period were extracted using a customized report designed for use in our EMR (Epic Systems Software, Inc., Verona, Wisconsin, USA). Data collected from EMR generated reports included demographic information such as age, timestamps for patient-care events, such as check-in-to-technician and check-in-to-provider times, and visit completion and check-out timestamps. A detailed review of in-person retina encounters for the month of April 2020 was compared with 2019 (21 working clinic days) to ascertain overall process time. The focus of this case study on the retina clinic during this month was due to the subspecialty’s sufficiently high patient volume and more urgent case-mix. Finally, we performed a retrospective review of 200 randomly selected charts for patients seen in the retina clinic within each period to detail changes in procedure volume. Main outcome metrics included the number of measurements of intraocular pressure (IOP) and number of dilations performed, as well as ancillary procedure volume, including utilization of ophthalmic imaging and intravitreal administration of medications.

### Timestamp calculations

Visit length was calculated by taking the time difference between the earliest and latest timestamps available for each patient’s visit. The earliest timestamp was typically “check-in time”. Of the two timestamps, “visit complete” and “visit complete, note pending,” the earlier was regarded as the end time of the visit. Check-in-to-technician time was used as a proxy for waiting room time for the simulation. Check-in-to-image-completion time was calculated by taking the time elapsed from a patient checking-in to the time of image completion. Time with technician was estimated by subtracting check-in-to-technician time from the earliest of the following timestamps: “In with technician,” “Done rooming,” “Dilating in waiting room,” or “Waiting for imaging.” For analysis, time spent in the provider phase of care was defined as the difference between check-in-to-provider time and total length of the visit. For the simulation, this number was halved to account for the fact that often two rooms were simultaneously assigned as “in with provider,” since no EMR-based timestamp delineates the actual location of the provider. Encounters with missing or erroneous timestamps were excluded from timestamp calculations but were still included in patient volume analyses.

### Assessment of patient satisfaction

The 15-item Medical Practice Survey was used to assess patient satisfaction (Press Ganey Associates, LLC) [13]. Outpatient discharge records from retina patients seen in-person in the clinic were randomly selected for postal mailings. The period of the COVID-related public health emergency (March 2020 to May 2020) was compared with the same period one year prior. Completed questionnaires were collected by mail, Internet and phone. The survey response format was Likert-type, on a scale from 1 to 5 as follows: Very Poor (1), Poor (2), Fair (3), Good (4), Very Good (5). A patient was considered satisfied with their experience in each category if they gave it a very good rating of 5. Scores of 1 through 4 were considered low satisfaction.

### Computer simulation model of clinic flow

To model clinic workflow, a discrete event simulation was utilized using Arena (Rockwell Automation, Wexford, Pennsylvania). EMR timestamp outcomes from retina visits in the month of April 2020 were used to set the variables in the simulation. Patients arrived on a schedule to simulate our retina clinic, which has 15-minute appointment blocks for 2-hours 45-minutes in the morning and afternoon, including a 1-hour 45-minute break without patient arrivals between sessions. The waiting room capacity was set at 10 people to reflect the need for spacing patients at least 6 feet apart. This was achieved by programming into the model a “hold queue” where patients would be placed before entering the simulated clinic, if the simulation detected a count of ten people within any of the wait-processes prior to being assigned a room. This count could include patient escorts or guests, which were estimated to arrive with one out of every fifteen patients. The guest limit per patient was set to a figure in-line with COVID-era guidelines [1]. There were two additional holds after the average waiting room time: one that scanned to make sure there was an empty room available, and another to make sure a technician was available. There was an additional three- to five-minute delay added to clean each exam room after it had been occupied. At a random point in the simulation, up to one “urgent” patient that skipped ahead in the arrival queues was included to simulate a disruption in the scheduled clinic flow.

The model was run for patient volumes of 10, 20, 25, 30, and 40 patients per provider, utilizing two different staff capacities: one provider with two technicians, and two providers with three technicians, reflecting the typical staffing of our retina clinic. These configurations will be referred to as the 1-provider and 2-provider models, respectively, each with 4 rooms available per provider. The following outcome measurements were generated by the model: simulated length of the clinic day, wait time experienced by patients before being placed in an exam room, total length of time each patient was in clinic, average length of time that the waiting room was at capacity, and maximum number of patients waiting to enter clinic during this period.

### Statistical analysis

Data were recorded in Microsoft Excel 2010 (version 14.0, Microsoft Corporation, Redmond, Washington) and analyzed using SPSS for Windows version 21.0 (SPSS Inc., Chicago, Illinois) and R software version 4.0.2 (R Project for Statistical Computing, Vienna, Austria; R Foundation for Statistical Computing; 2019, Available at: https://www.r-project.org). A chi-square test with Bonferroni correction was used for between-group analysis. The Z-test was used to determine the difference between the mean top box response rates of respondents on patient experience surveys. Clinic-timing variables as well as averages generated in 1-provider and 2-provider models were compared using independent t-tests. All tests were 2-sided and *p* values below 0.05 were considered statistically significant.

## Results

**Fig. 1:**
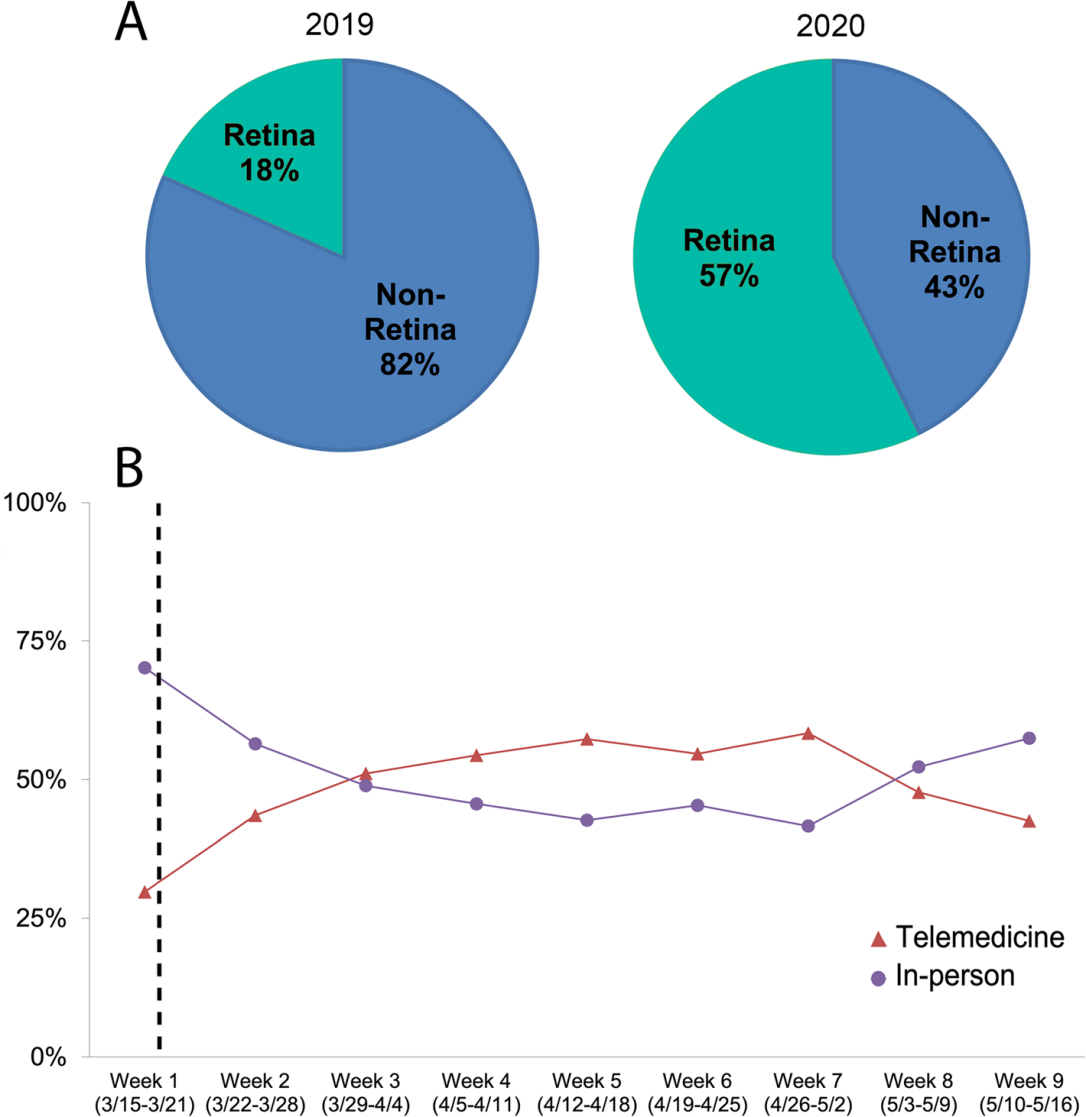
In-person and telehealth clinic encounters between March 15th and May 15th in 2019 and 2020. **a** In-person clinic encounters in 2019 and 2020. In 2019, 18 % of all in-person visits were retina encounters (*n* = 2,647) whereas in 2020 that proportion rose to 57 % (*n* = 1,015). **b** In-person and telemedicine encounters in 2020. In 2020, the retina service saw an increase in the proportion of telehealth visits relative to in-person encounters. Whereas in 2019 telehealth visits did not occur at all, during the peak pandemic period in Massachusetts, telehealth was being leveraged in over 50 % of all retina patient encounters on a weekly basis. Dashed vertical line represents the start of the Massachusetts stay-at-home order on March 16, 2020.

In comparing the period of the COVID-19 health emergency from March 15 to May 15 in 2020 to the same period one year before, the total number of in-person ophthalmology visits fell by 88 % from 14,486 to 1,774 visits. Similarly, visits to the retina clinic declined by 62 % from 2,647 to 1,015. Whereas retina visits previously accounted for only 18 % of total ophthalmology visits, within the COVID-period they increased to more than 57 % of in-person appointments (χ^2^ = 1363, *p* < 0.0001) (Fig. 1 a). Telemedicine visits, which were not previously utilized in our practice, accounted for 77 % of all ophthalmology encounters during this period in 2020, and represented 49 % of retina encounters (Fig. 1b).

Although there was no difference in the average age of patients treated by the retina service during the period of the COVID-19 health emergency in 2020 compared with the same period in 2019 (74.7 ± 13.6 years versus 74.3 ± 13.6 years, *p* = 0.490), patients seen in-person in the retina clinic in 2020 were older compared with those seen in-person in 2019 (76.8 ± 13.4 years versus 72.5 ± 13.5 years, *p* < 0.0001) (Supplemental Fig. 1A). In contrast, retina patients treated by telehealth in 2020 were younger than those patients seen in clinic during the same period in 2020 (72.5 ± 13.5 years versus 74.7 ± 13.6 years versus, *p* < 0.0001) (Supplemental Fig. 1A). Additional patient demographic and clinical characteristics are presented in Table 1.

**Table 1:** Patient demographics and average timestamp values in 2019 and 2020

Age (years)	Mean (SD)	Median	Min	Max	*p*-value
2019 (in-person)	74.3 (13.6)	75	22	107	
2020 (in-person)	76.8 (13.4)	79	22	102	**< 0.001**
2020 (telehealth)	72.5 (13.5)	73.5	28	102	**< 0.001**
**Check-in to tech time (min)**					
2019	14 (15)	10	0	213	
2020	3 (4)	2	0	47	**< 0.001**
**Check-in to image completion time (min)**					
2019	45 (25)	40	10	275	
2020	31 (16)	29	2	133	**< 0.001**
**Check-in to provider time (min)**					
2019	69 (30)	66	3	264	
2020	33 (18)	30	3	95	**< 0.001**
**Total Visit Length (min)**					
2019	87 (35)	82	17	298	
2020	47 (33)	40	4	285	**< 0.001**

### Retina clinic analysis

During the peak of the COVID-19 health emergency in April 2020 retina clinic in-person patient volume declined by 66 % compared with one year prior (425 visits versus 1,245 visits). Imaging fell by more than 90 %, occurring in only 24 % of visits in 2020, compared with 83 % of retina visits in 2019 (*p* < 0.0001). The average number of patients seen per retina provider per day also declined by more than 50 % to 11.8 ± 1.9 patients (median 12, range of 9 to 14 patients) in 2020, compared with 26.6 ± 3.2 patients (median 28, range of 21 to 29 patients) in 2019 (*p* < 0.0001).

**Fig. 2:**
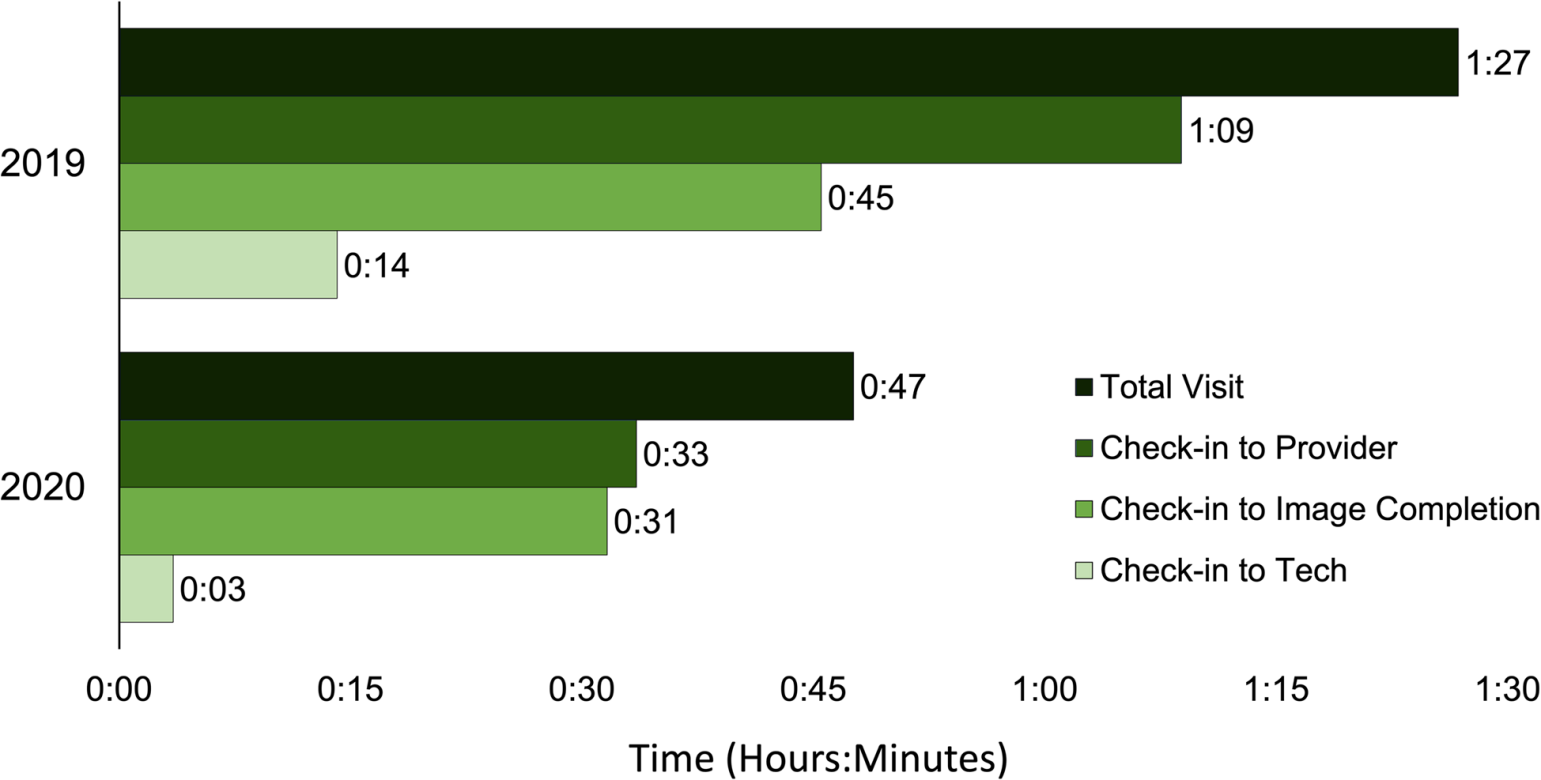
Average in-person encounter task times. Average visit length declined by 46 % from 87 to 47 min. Check-in to provider time declined by 52 % from 69 to 33 min. Check-in to technician time declined by 79 % from 14 to 3 min. Check-in to image completion time declined by 31 % from 45 to 31 min.

The retina service also experienced changes in both procedural volume and visit length during the COVID-19 health emergency. The percentage of encounters with a documented IOP measurement declined from 97 to 56 % (*p* < 0.0001). Whereas dilation occurred in 87 % of encounters in 2019, dilation was performed in only 19 % of eyes during the same period in 2020 (*p* < 0.0001). Both patients’ eyes were dilated for 62 % of encounters in 2019, while in 2020 this occurred in fewer than 6 % of visits (*p* < 0.0001). The percentage of visits with one eye dilated declined from 25 to 3 % (*p* < 0.0001). In contrast, the percentage of retina patients undergoing intravitreal injection of medications rose from 49 % of all encounters in 2019 to 86 % of clinic visits in 2020 (*p* < 0.0001), and the percent of patients undergoing bilateral injections rose from 9 to 17 % (*p* = 0.09). The proportion of patients undergoing OCT imaging declined from 71 to 19 % (*p* < 0.0001), and the proportion of encounters with imaging other than OCT remained similar, occurring in 4 % of retina visits in 2019 compared with 5 % in 2020 (*p* = 0.733). The average length of the visit to the clinic declined by 46 %, to 47 ± 33 min in 2020 compared with 87 ± 35 min in 2019 (*p* < 0.001). Time spent in the physician phase of care declined 53 % from 38 ± 33 min to 18 ± 22 min (*p* < 0.0001). Similarly, retina service check-in to technician, check-in to provider, and check-in to image completion times declined by 79 %, 52 %, and 31 %, respectively (Fig. 2; Table 1).

### Impact of COVID-19 on patient satisfaction

Although significantly fewer outpatient in-person visits took place during the months encompassing the COVID-related health emergency, there was no difference in patient experience survey return rate compared with the same period one year prior (23.8 % versus 26.1 %, *p* = 0.418). The impact of COVID-related changes to clinic flow had noteworthy impacts on patient satisfaction related to patient access, moving through the visit, and interpersonal communication between patient and nursing assistant and/or provider **(**Table 2**)**. Patients rated the ease of contact (email, phone, and web portal) at 84.6 % during the COVID-related health emergency compared with 61.0 % in the same months one year earlier (*p* = 0.001). Satisfaction with the degree to which patients were informed about delays (71.8 % versus 33.3 %, *p* < 0.001) and wait time at the clinic (68.4 % versus 27.6 %, *p* < 0.001) also more than doubled during the COVID-period compared with the same period one year earlier. Ratings related to the provider’s concern for questions or worries, explanations about problems or conditions, and effort to include the patient in decisions also all rose, albeit to a lesser extent compared with the pre-COVID period (Table 2). Finally, patient overall rating of “How well the staff worked together to care for you” rose to 95.1 % from 75.0 % one year prior (*p* = 0.008), paralleling nearly as steep of a rise in the “likelihood of patients to recommend the retina practice overall” (78.3 % versus 93.2 %, *p* = 0.038).

**Table 2:** Patient satisfaction results for patients seen in the retina clinic

	COVID-related Health Emergency^1^	Pre-COVID Period^2^	*Z-score*	*p-value*
**ACCESS**				
Ease of scheduling your appointment:	88.6 %	61.0 %	3.116	**0.002**
Ease of contacting (e.g., email, phone, web portal):	84.6 %	n/a^3^		
**MOVING THROUGH YOUR VISIT**				
Degree to which you were informed about any delays:	71.8 %	33.3 %	3.616	**< 0.001**
Wait time at clinic (from arriving to leaving):	68.4 %	27.6 %	3.944	**< 0.001**
**NURSE/ASSISTANT**				
How well the nurse/assistant listened to you:	88.9 %	n/a^3^		
Concern the nurse/assistant showed for your problem:	78.8 %	60.4 %	1.771	0.076
**CARE PROVIDER**				
Concern the care provider showed for your questions or worries:	93.8 %	74.1 %	2.676	**0.007**
Explanations the care provider gave you about your problem or condition:	89.4 %	72.9 %	2.114	**0.034**
Care provider’s effort to include you in decisions about your care:	91.1 %	74.1 %	2.118	**0.028**
Care provider’s discussion of any proposed treatment (options, risks, benefits, etc.):	86.7 %	n/a^3^		
Likelihood of your recommending this care provider to others:	87.2 %	80.4 %	0.936	0.347
**PERSONAL ISSUES**				
Our concern for your privacy:	88.1 %	81.7 %	0.878	0.378
How well the staff protected your safety (by washing hands, wearing ID, etc.):	92.5 %	79.7 %	1.748	0.080
**OVERALL ASSESSMENT**				
How well the staff worked together to care for you:	95.1 %	75.0 %	2.645	**0.008**
Likelihood of your recommending our practice to others:	93.2 %	78.3 %	2.073	**0.038**

^1^Patient experience returns[8] for the three months during the peak of the COVID-related public health emergency (March 2020 to May 2020)

^2^Patient experience returns one year prior to the COVID-related public health emergency (March 2019 to May 2019)

^3^The 15-question revised Medical Practice Survey (2019) added several questions to better measure certain aspects of the patient overall experience, as well as other minor changes to the instrument.^16^ For comparison purposes, in the two months preceding the COVID health emergency, “Ease of contacting” rose from 61.6 % (*p* = 0.012), “How well the nurse/assistant listened” rose from 69.6 % (*p* = 0.031) and “Care provider’s discussion of any proposed treatment” remained unchanged from 83.9 % (*p* = 0.701)

Several aspects of the patient experience were largely unaffected by the COVID-related changes to care. Concern for both privacy and safety remained highly rated by patients in both periods, and although a slightly higher number of patients expressed confidence in how these aspects of their visits were handled during the COVID-19 health emergency, the change did not reach statistical significance (Table 2). Recommendation of the retina care providers was also largely unaffected by the COVID-related changes (87.2 % versus 80.4 %, *p* = 0.347).

### Discrete‐event simulation modeling

**Fig. 3:**
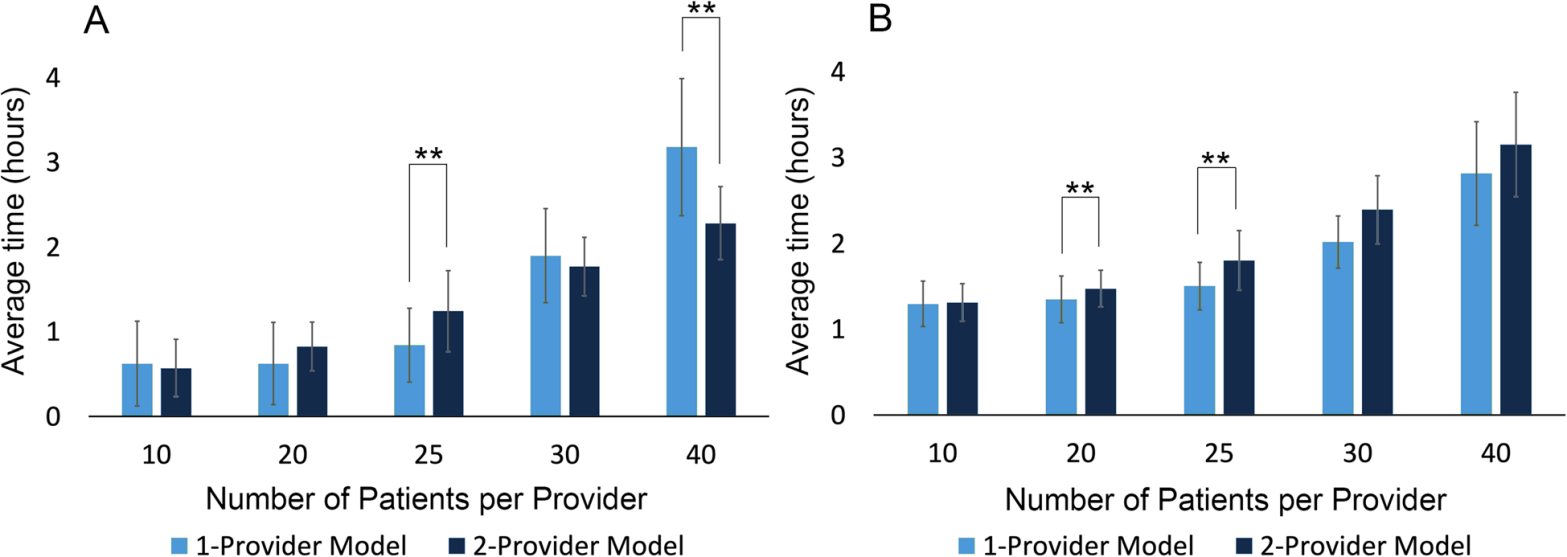
Simulated patient time in clinic. **a** Average total wait time before each patient was roomed in both the 1-provider and 2-provider models. **b** Average visit length in both the 1 provider, 2 technician and 2 provider, 3 technician models. Error bars present 95 % confidence intervals. (** *p* < 0.01).

The EMR timestamp results were used to set the following variables in our model: waiting room time (4 ± 5 min), time with technician (19 ± 14 min), examination time (8 ± 6 min), percent of patients requiring dilation (19 %), and percent of patients receiving imaging (24 %). The simulated length of the clinic day increased with increasing patient volumes, reaching a maximum of nearly 12 h at 40-patients-per-provider in both the 1- and 2-provider models (11.5 ± 0.45 h and 11.9 ± 0.36 h, respectively). There was no significant difference in average clinic day length between the 1-provider and 2-provider models at any of the patient volumes tested. At 25-patients-per-provider, the wait time before an arriving patient was assigned to a room became significantly longer in the 2-provider model, compared with the 1-provider model (50.4 ± 20.1 min versus 74.7 ± 28.6 min, *p* = 0.008). Above this patient volume, it took longer for patients to be assigned to a room in the 1-provider model, compared with the 2-provider model, reaching a significant difference at a patient volume of 40 patients (190.7 ± 48.5 min versus 136.9 ± 25.8 min, *p* < 0.001) (Fig. 3 a). The average total length of the visit per patient increased with increasing patient volumes and was significantly higher at a patient volume of 25-patients-per-provider in the 1-provider model (108.1 ± 20.8 min versus 90.1 ± 16.7 min, *p* = 0.005) and 30-patients-per-provider (143.4 ± 23.7 min versus 120.9 ± 18.3 min, *p* = 0.002) in the 2-provider model (Fig. 3b). At patient volumes above 25-patients-per-provider, the waiting room was above clinic-defined capacity for significantly longer periods of time in the 2-provider model, compared with the 1-provider model (Table 3).

**Table 3:** Average time the waiting room was at capacity and maximum number of people waiting across increasing patient volumes and provider capacities

	Average time at capacity (minutes)			Maximum # patients waiting to enter (%)		
Volume (patients)	**1-Provider**	**2-Provider**	***p*-value**	**1-Provider**	**2-Provider**	***p*-value**
10	0	0	-	0	0	-
20	< 1	< 1	0.222	2 (10 %)	4 (10 %)	0.156
25	0	3	**0.023**	0	8 (16 %)	**0.014**
30	< 1	18.6	**< 0.001**	2 (6.7 %)	21 (35 %)	**< 0.001**
40	10.04	59.8	**< 0.001**	12 (30 %)	36 (45 %)	**< 0.001**

## Discussion

In response to the COVID-19 pandemic, public health recommendations and national guidelines resulted in a nationwide, temporary reduction in in-person eye care [4, 9]. Within the first six weeks after the announcement of the state-wide stay-at-home order in Massachusetts [4], total ophthalmology volume at our academic multispecialty group practice fell significantly [1]. This decrease in clinical volume made better physical distancing possible between patients and staff, and it freed up much needed physical space to implement updated patient care protocols. Our study reports the impact of these changes in the retina clinic during the period when this service provided uninterrupted, essential care to a large number of patients despite the public health emergency. Our results show that eye care can be delivered while maintaining COVID-era precautions and that patient satisfaction was enhanced by these measures. Our study is also among the first to use EMR-timestamped reports to study the clinical transformation, efficiency, and patient flow changes to outpatient systems during the COVID-era. While other fields of medicine such as gastroenterology, cardiology and nephrology utilized discrete-event simulation to study changes in operations during the COVID-19 period [12–16], the present study is the first to do so in ophthalmology. Using EMR-derived timestamps from actual patient encounters, we present a discrete-event simulation model capable of extrapolating from clinic-level data the impact of COVID-era changes on patient flow with the aim of planning for future patient volume increases. This is relevant because many of the measures instituted in response to the pandemic are likely to remain in place for the foreseeable future. Furthermore, the dynamic nature of the model allows for emerging changes to be simulated before actually being deployed in the clinic.

The retina service underwent a major transformation in the way care was delivered during the early stages of the pandemic compared with the same time period in 2019. As a result of intentionally reducing patient and procedural volume, the retina service achieved significant reductions in the average length of visit, check-in to technician time, image completion time, and time in the provider phase of care. Although other studies also demonstrated similar changes, including decreased patient and procedural volume, they did not study the impact of such changes on clinic time [17]. The improved patient experience was also reflected by increased satisfaction with being provided information about delays and total length of the visit. By contrast, the largely unaffected likelihood of patients being willing to recommend their care providers reflects the enduring nature of the patient-physician relationship, which was unaffected by these changes made in response to COVID-19. However, only patients with relatively urgent or emergent conditions were granted access to the clinic during the period of the COVID-19 health emergency, and this restriction to a subset of patients may bias our patient satisfaction results. Other studies have demonstrated that pandemic-related postponement in patient care was significantly associated with worse short-term outcomes [18, 19]. We note that nearly half of retina visits were conducted by telehealth during this period and that patient satisfaction with both access and the provider were rated at similarly high levels for telehealth encounters compared with in-person encounters (data not shown). We did not study clinical outcomes. However, our findings suggest that outcomes were likely favorable for the patients who used telehealth services compared to in-person services because patients were similarly satisfied with the care and access they received. No patient satisfaction data exist to compare patient experience with telehealth during the COVID-19 health emergency with the period before, as telehealth visits did not occur in 2019. Other studies have reported findings on the acceptance of telemedicine by patients in ophthalmology [20–22]. These studies found that a wide range of acute and routine diagnoses were managed via video visits and patients rated their experiences highly and would consider using such consultations into the future [20–22]. The fact that COVID-era changes are likely to remain, especially those pertaining to telehealth, supports the use of a dynamic modeling system, such as the one we used in this study, to further help predict and support clinic operational changes into the future.

To increase clinic throughput during the outbreak of COVID-19, the eye exam was streamlined, and ancillary testing was either reduced or performed at different times [1]. These changes decreased the amount of time that each patient spent in the clinic, facilitating physical distancing. Previously, it was common to dilate both eyes (62 %), whereas during this period, one or both eyes were dilated only 19 % of the time. This reflects the relatively greater proportion of problem-focused visits conducted during the COVID-period, since routine retinal exams were postponed [1, 7]. The proportion of encounters with imaging also declined significantly between the two periods. Although the number of OCT studies declined precipitously during the COVID-period, the rate of other types of imaging studies, e.g., fluorescein angiograms, performed relative to the number of visits remained unchanged compared with the same period one year earlier. This finding indicates that when diagnostic imaging was required, the service was still able to be delivered, similar to findings suggested by other studies of ophthalmology practice changes during the COVID-era where imaging was only done when diagnostically critical during the COVID-19 period [11].

In parallel, the proportion of patients undergoing intravitreal injections of medications significantly increased during the COVID-era in this study. This increased proportion, given the decline in other eye care services noted above, suggests the need for treatment with an intravitreal agent was the most likely reason for a patient to be seen in the retina clinic during the peak of the COVID-19 public health emergency in our region. Interestingly, other studies also revealed decreases in the overall number of intravitreal injections performed during the COVID-19 period compared to one year prior. One study found a 50 % reduction in injections using a chart review [23], while another found only a 9.9 % absolute reduction [24]. In our study, a greater proportion of patients undergoing treatment with intravitreal injections increased the productivity per retina visit during this period, thereby at least partially offsetting some of the loss of clinic volume. A larger proportion of patients also underwent bilateral injections in 2020 compared with 2019 in our study. One of the likely reasons is that patients elected to accept bilateral injections in an effort to make fewer visits to the clinic. Whether this change was driven by patient or provider preference cannot be determined from the evidence available, but the net effect was a further reduction in the number of in-person appointments. The impact of these changes on the effectiveness of care delivered and patient outcomes, if any, remains to be determined.

The mean age of patients attending the retina clinic in 2020 was older than in 2019 and higher compared with 2020 telemedicine encounters. This is likely because older patients more often have serious eye conditions that require immediate, in-person attention compared to younger patients. Further, older patients are more likely to have conditions that require treatment with intravitreal injections of medication. Older patients may also be generally less comfortable with telemedicine visits, preferring in-person care. However, providers played a leading role in deciding which patients were to have in-person visits. Another large study assessing the effectiveness of their virtual ophthalmic care found the median age of their virtual visit patients to be 32 years old, which was significantly younger than that of face-to-face patients at 45 years old [25]. This study found that retinopathy was the most common reason for in-person visits, similar to our study, while ocular surface diseases were most cited in virtual appointments [25]. This suggests that telemedicine visits may be used to effectively triage patient complaints.

Discrete-event simulation provides a model of the operations of a system in sequence, and it has been used extensively as a tool for analyzing healthcare systems with a goal of quality improvement [26]. In ophthalmology, discrete-event simulation has been effectively employed to model outpatient clinic flow and capacity restraints [27–30], and results have been validated by the inclusion of time-stamped EMR data [31]. Our model sought to assess the impact of measures adopted after the outbreak of COVID-19 on retina clinic operations. Some of the changes included limiting the waiting room capacity to maintain at least a six-foot separation between patients, restricting patients to a single exam room rather than moving patients between multiple technician and provider rooms, and ensuring that check-in and check-out processes allow for adequate social distancing. Our model provides insight into potential bottlenecks created by these changes. Finding alternative spaces to stage patients will be an important strategy, especially during months of the year when it is less practical to recommend that patients wait outside of the clinic, for example, in their personal vehicles [1]. The model also found that at the highest patient volumes, patients waited less time to be roomed in the 2-provider model but spent the same amount of total time in the clinic compared with the 1-provider model. This reveals that the bottleneck is not attributable to the COVID-19 rooming system per se, but rather to provider availability at higher patient volumes. In the future, it may even be possible to run the model in conjunction with actual operation of the clinic to adjust dynamically resource allocation, e.g., reassigning rooms or technical staff among providers. This information could also enable the scheduling staff to communicate projected delays to patients who have yet to arrive, move patients to another provider’s schedule, or even preemptively reschedule visits in response to the volume of patients and wait times. Finally, the model can also be used to assess utilization of clinic capacity, thereby informing scheduling decisions related to the ratio of patients to providers or other types of staff in order to optimize clinical productivity.

There are limitations to this type of study. Foremost is that time-stamped data depends on user input for consistency, and in our study, in-person observation or video recording was not available to validate our internal timestamps for accuracy [31]. Out of 1670 patient encounters examined, all but one had one or more time-stamped phases of care. However, in total, 36 % of visits had at least one missing timestamp, and the omission rate was substantially greater in the COVID-19 period compared with the period one year prior (82 % versus 21 % of encounters, *p* < 0.001). As the recording of these events happen behind the scenes in the EMR, this suggests that staff may have been less accurate in documenting changes in the phases of care during the period of COVID-19 as they moved through the visit. Another limitation inherent to our model is that it uses assumptions based on EMR timestamps and event rates derived from a period when the clinic was far below historical utilization rates and seeing far fewer patients. Although this period reflects the completely redesigned patient flow and practice measures that reflect COVID-era restrictions, it remains to be seen if all of the assumptions made in the model hold as the clinic returns to full capacity. When more patients start to return for routine visits, the proportion of eyes being dilated, receiving IOP checks, and/or being imaged is likely to increase. In keeping with changes in these rates, it will be necessary to re-run the model, which will increase the accuracy of predictions for clinic operations. Finally, it will be important to assess if patient satisfaction levels are maintained as the number of patients increases and more routine care is provided.

## Conclusions

 During the COVID-19 stay-at-home order, ophthalmology and, specifically, the retina service saw major shifts in the way eye care was delivered. Maintaining these improvements in performance, enhanced cleaning, and social distancing policies will pose a significant challenge when the demand for eye care increases. Discrete-event simulation models are an important strategy to allow practices to plan for volume increases while maintaining a safe clinical environment. Our findings support the conclusion that an ophthalmology practice is highly adaptable. The changes implemented enhanced the effective delivery of eye care and improved the patient’s sense of well-being, thus potentially becoming a new standard of care.

## Supplementary information

Additional file 1: **Figure S1.** Density plot of the age distribution of retina patients. **a**Patients seen in-person in the retina clinic in 2020 were older compared with those seen in-person in 2019. **b** In contrast, retina patients treated by telehealth in 2020 were younger than those patients seen in clinic during the same period in 2020.

## Data Availability

The datasets generated and/or analyzed during the current study are not publicly available due to the use of internal and confidential patient medical record data stored on internal, confidential and protected hard drives, but de-identified and redacted data are available from the corresponding author on reasonable request.

## Abbreviations

COVID-19: coronavirus disease 2019
SARS‐CoV‐2: severe acute respiratory syndrome coronavirus 2
IOP: intraocular pressure
EMR: electronic medical record
OCT: optical coherence tomography
