# Supplementary material for: Modeling the impact of COVID-19 on Retina Clinic Performance
Source: BMC Ophthalmol. 2021 May 10;21:206. doi: 10.1186/s12886-021-01955-x (PMC8107774; doi:10.1186/s12886-021-01955-x)
Supplement: Supplementary file 1 — Figure S1. Density plot of the age distribution of retina patients. aPatients seen in-person in the retina clinic in 2020 were older compared with those seen in-person in 2019. b In contrast, retina patients treated by telehealth in 2020 were younger than those patients seen in clinic during the same period in 2020. [file 12886_2021_1955_MOESM1_ESM.pdf]

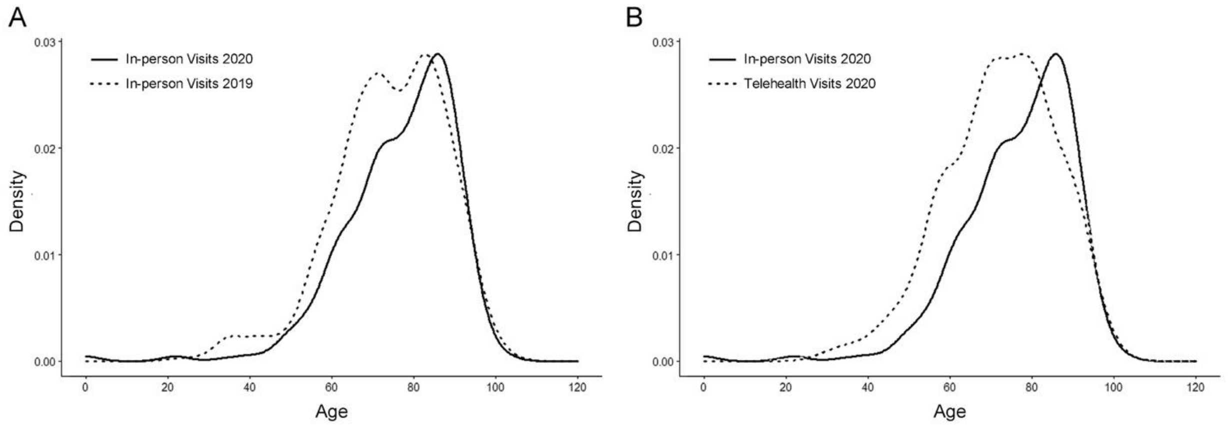

Supplemental Figure 1. Density plot of the age distribution of retina patients. (A) Patients seen in-person in the retina clinic in 2020 were older compared with those seen in-person in 2019. (B) In contrast, retina patients treated by telehealth in 2020 were younger than those patients seen in clinic during the same period in 2020.
